# Osseous Union of Temporary Teeth

**Published:** 1892-12

**Authors:** W. A. Robertson

**Affiliations:** Cookston, Minn.


ARTICLE IV.

### OSSEOUS UNION OF TEMPORARY TEETH.

BY W. A. ROBERTSON, D. D. S., COOKSTON, MINN.

While fusion of the teeth is uncommon, specimens are to be met with in almost every dental collection, showing that it occurs often enough to make it of practical importance to the dental practitioner.

Having just come into possession of an interesting specimen of this class, I thought it might be of interest to some of your readers to have it described. This specimen is of particular interest from the fact that one of the teeth is a supernumerary, and as the teeth belong to the temporary set, it is of somewhat rare occurrence, so that a short history of the case may not be amiss.

About three years ago the patient was brought to us by his mother to have some filling done on his front teeth. We were struck by the fact that there were five superior incisiors, there being three on the left side, two of which overlapped and were decayed. Upon preparing them for filling we found they were united, and drew the attention of the mother to this fact, at the same time asking her to save them for me when they were taken out. A few weeks ago they became so loose and sore that the application of a thread was all that was necessary to accomplish their removal.

Upon close examination there is perfect union of the roots, one of which is considerably absorbed while the other is almost entire. There is a shallow groove in front and a deeper one behind, showing the point of union of the roots. The fusion is perfect from the cervical margin to the apex of the root, and has the appearance of having originally included the crowns as well, but owing to the enamel being underminded by decay and broken away, it is not complete now.

In a case like this we might expect to find an extra permanent incisor, on the ground of the accepted theory of development of the permanent teeth, viz., from the cords of the temporary teeth, and we will watch this case with interest on that account, and report if such is the case.

The teeth are both well developed and are almost uniform in size, so that it is impossible to say which is the supernumerary.

There is described in the American System of Dentistry (page 419, Vol. III., fig. 115), a case very nearly similar, and the cut conveys a good idea of this one except that there are three teeth in place of two. It is from the collection of Dr. Douglas, Rosino, Michigan, and is the only one I can find recorded of union between a supernumerary and central incisor of the temporary set.—*Dominion Dental Journal.*

